# Prostaglandins in breast cancer: relationship to disease stage and hormone status.

**DOI:** 10.1038/bjc.1983.251

**Published:** 1983-11

**Authors:** R. A. Karmali, S. Welt, H. T. Thaler, F. Lefevre

## Abstract

Tissue prostaglandin (PG) content and production by human breast cancers were measured in 24 human mammary carcinoma specimens. The 5 compounds studied were PGE1, PGE2, PGF2 alpha, 6-keto-PGF1 alpha, and TXB2. The tissue content of all 5 compounds was higher in neoplastic tissue in comparison with the paired noncancerous breast tissue. However, microsomal PG synthetase activity in vitro in noncancerous and neoplastic breast tissue was comparable. Increased thromboxane formation was associated with three clinical variables--tumour size, axillary lymph node metastases and distant metastasis. A lesion negative for either oestrogen or progesterone receptor content tended to produce more TXB2 but lower PGE2 and 6-keto-PGF1 alpha. Results obtained in this pilot study may provide clues as to what direction future larger studies could take in the search for reliable prognostic indicators for breast cancer.


*Br. J. Cancer* (1983), **48**, 689–696

### Prostaglandins in breast cancer: Relationship to disease stage and hormone status

R.A. Karmali, S. Welt, H.T. Thaler & F. Lefevre

*Memorial Sloan-Kettering Cancer Center, 1275 York Avenue, New York, NY 10021, U.S.A.*

**Summary** Tissue prostaglandin (PG) content and production by human breast cancers were measured in 24 human mammary carcinoma specimens. The 5 compounds studied were $PGE_1$, $PGE_2$, $PGF_{2\alpha}$, 6-keto-$PGF_{1\alpha}$, and $TXB_2$. The tissue content of all 5 compounds was higher in neoplastic tissue in comparison with the paired noncancerous breast tissue. However, microsomal PG synthetase activity *in vitro* in noncancerous and neoplastic breast tissue was comparable. Increased thromboxane formation was associated with three clinical variables—tumour size, axillary lymph node metastases and distant metastasis. A lesion negative for either oestrogen or progesterone receptor content tended to produce more $TXB_2$ but lower $PGE_2$ and 6-keto-$PGF_{1\alpha}$. Results obtained in this pilot study may provide clues as to what direction future larger studies could take in the search for reliable prognostic indicators for breast cancer.

The evidence linking prostaglandins (PGs), particularly of the E series, to human mammary cancer is substantial, and this topic has been reviewed previously (Bennett, 1979; Karmali, 1980). In this disease excess prostaglandin E production by tumours appears to be responsible for occasional cases of hypercalcaemia (Seyberth *et al.*, 1975) and may contribute to the development of metastatic disease (Rolland *et al.*, 1980). The survival time after mastectomy was found to correlate inversely with amounts of "prostaglandin-like" material extracted from human mammary cancers (Bennett *et al.*, 1979). The ever-increasing list of references presenting evidence of enhanced PG content and synthetic capacity in many human tumours of different types therefore makes the reasons for continuing to study PGs in cancer compelling.

Recent studies suggest that the many physiological and pathological effects formerly attributed to the classical PGs, namely, $PGE_2$ and $PGF_{2\alpha}$, may in fact be attributable to the action of other oxygenated metabolites of arachidonic acid, including thromboxanes, prostacyclin and other potentially important prostanoids.

We report here a comparison of tissue content and biosynthesis of $PGE_1$, $PGE_2$, $PGF_{2\alpha}$, 6-keto-$PGF_{1\alpha}$ (a stable degradation product of $PGI_2$) and $TXB_2$ (a stable degradation product of thromboxane $A_2$) in a series of 24 human mammary carcinomas. PG and $TXB_2$ production in each specimen was examined in relation to tumour size, axillary lymph node metastases, distant metastasis and hormone status (oestrogen and progesterone).

Correspondence: R.A. Karmali.

Received 17 May 1983; accepted 1 August 1983.

### Materials and methods

The study concerns 24 patients referred to the Memorial Sloan-Kettering Cancer Center, New York for evaluation and treatment of breast masses. All biopsies, primary surgical procedures, pathological evaluation and procurement of the specimens were made at the Center. Tissues for prostaglandin studies were obtained immediately after the patient underwent the initial surgical procedure. Approximately 2 g each of the tumour mass and its paired noncancerous ductal tissue from the same specimen were delivered promptly to the laboratory by staff of the Tumour Procurement Center. Analyses of prostaglandin yields (tissue content plus any metabolism occurring during the processing procedures) and prostaglandin synthetase activity were carried out immediately or after storage at −70°C.

The patients underwent initial clinical staging and all abnormalities were further evaluated with X-rays, radionuclide scans and, if necessary, from biopsies. The clinical and pathological staging accorded with the Classification of the American Joint Committee for Cancer Staging (1979). The results are shown in Table I. All were infiltrating ductal adenocarcinomas except for patient No. 20, who had invasive lobular carcinoma. Histology of the noncancerous control specimens showed no evidence of chronic mastitis, fibrocystic disease or presence of inflammatory cells, and were reported to be normal.

© The Macmillan Press Ltd., 1983

**Table I** Clinical information about 24 breast carcinomas[a, b]

| *Patient* | *Age* | *Oestrogen receptor*[c] | *Progesterone receptor*[c] | *Tumour size (largest diameter) cm* | *No. of +ve/ total lymph nodes* | *Distant metastases bone, liver or lung* | *Stage* |
|---|---|---|---|---|---|---|---|
| 1 | 60 | + | + | 3 | 0/23 | | II |
| 2 | 50 | | | 19 | 15/31 | + | IV |
| 3 | 67 | + | + | 4 | 14/15 | + | IV |
| 4 | 59 | + | | 6.5 | 0/23 | | III |
| 5 | 52 | | | 6.5 | 4/19 | | III |
| 6 | 45 | | | 5.5 | 10/30 | | III |
| 7 | 60 | + | + | 2.2 | 0/32 | | II |
| 8 | 55 | | | 12 | 12/12 | + | IV |
| 9 | 57 | + | + | 4 | 3/25 | | II |
| 10 | 56 | | | 4 | 15/16 | + | IV |
| 11 | 72 | | | 8 | 31/31 | + | IV |
| 12 | 36 | | | 4.8 | 0/32 | | II |
| 13 | 67 | + | | 1.6 | 0/22 | | I |
| 14 | 54 | | | 5 | 10/27 | + | IV |
| 15 | 39 | + | | | 1/28 | | II |
| 16 | 73 | | | 3.5 | 14/18 | | IV |
| 17 | 38 | | | 11 | 17/18 | + | IV |
| 18 | 49 | + | + | 3 | 0/22 | | II |
| 19 | 44 | + | | 3.5 | 1/20 | | II |
| 20 | 69 | + | + | 3 | 2/25 | + | IV |
| 21 | 51 | | | 8 | 4/27 | | IV |
| 22 | 31 | | | 8.5 | 10/19 | | III |
| 23 | 52 | | | 5.5 | 2/21 | | II |
| 24 | 71 | + | | 3 | 5/15 | | II |

[a]The 24 cases of human mammary cancers in this study were patients referred for evaluation and treatment to the Memorial Sloan-Kettering Cancer Center, New York, N.Y.
[b]Post-surgical treatment pathology classification of the UICC-AJC 1977.
[c]The cutoff point at which the specimen was considered positive for oestrogen or progesterone was 6 fmol $mg^{-1}$ protein.

*Prostaglandin measurement procedures*

*Materials* Prostaglandin standards $PGE_1$, $PGE_2$, $PGF_{2\alpha}$ tromethamine salt, 6-keto-$PGF_{1\alpha}$ and $TXB_2$ were kindly supplied by Dr. J. Pike (Upjohn Company, Kalamazoo, Michigan). Tritium-labelled compounds were purchased from New England Nuclear (Boston, Massachusetts). Rabbit antisera to $PGE_1$ and $PGE_2$ were obtained from the Pasteur Institute (Paris, France). Antibodies to $PGF_{2\alpha}$, 6-keto-$PGF_{1\alpha}$ and $TXB_2$ were raised in our laboratory (R.K.). The cross-reactivities of these antibodies for the nontargeted PGs were no greater than 4% except for $PGE_1$ and $PGE_2$ antisera which cross-reacted 10% with $PGE_2$ and $PGE_1$ standards, respectively. Unlabelled arachidonic acid (Grade 1) was obtained from Sigma (St. Louis, Missouri).

*Analytical methods—Extraction* The procedure for extracting the prostaglandins was described earlier (Karmali *et al.*, 1982). Briefly, a trace of [$^3$H]-PG was added to aliquots of standards and samples before being extracted once with 3.5 ml petroleum ether. After acidification to pH 3.5, the samples were extracted twice with diethyl ether, dried under nitrogen and reconstituted in assay buffer. The efficiency of the extraction procedure to this point was 85–95%.

*Radioimmunoassay* (*RIA*) Standard quantities of each prostaglandin (0–1000 pg) or the extracted sample to be measured were prepared in 0.1 ml aliquots of assay buffer. Antiserum and label were added successively in 0.1 ml aliquots and incubated at 4°C for 8–12 h. Bound and free [$^3$H]-PG were separated by 0.5 ml dextran-coated charcoal (0.5–1.0% by wt) to estimate the amount of each compound in the unknown samples.

The sensitivity of the assays has been found to be $\sim$10 pg. The intra-assay coefficient of variation was 9.0%.

*Assay of prostaglandin-like material extracted from mammary tissue* Solid tumour fragments weighing between 0.5 and 1 g were ground using a mortar and pestle at 2°C in a 1:5 (g:ml) tissue:buffer volume of MES buffer (1 M 2n-morpholino-ethane sulphonic acid, pH 7.4, containing 2 mM $CaCl_2$, 2% glycerol and 1 mM monothio-glycerol). No antioxidant was added to avoid altering the PG synthetase. Homogenates were centrifuged at 800 *g* for 15 min at 4°C. Each supernatant thus obtained was further spun at 150,000 *g* for 1 h at 4°C and stored in duplicate at −20°C. After adding [$^3$H]-$PGE_2$ as a tracer, the PGs were extracted and measured by RIA as described above. Such measurements represent the yield of the 5 immunoreactive compounds in noncancerous and tumour tissues. The remaining microsomal pellet obtained after centrifugation at 150,000 *g* was saved for prostaglandin synthetase studies.

*Prostaglandin synthetase assay* The microsomal pellet was suspended in MES buffer; its protein content was measured by the Lowry method and adjusted to 0.5 mg protein $ml^{-1}$ in MES buffer. Biosynthesis of PGs by microsomal preparations of noncancerous and neoplastic mammary tissue was assayed by a modification of the procedure described by Rolland *et al.* (1980).

A 0.2 ml aliquot of the microsomal fraction was incubated at 37°C with 0.8 ml MES buffer containing 1.25 mM reduced glutathione, 1.25 mM adrenaline, and 1.25 μM sodium arachidonate. After incubation for 10 min, [$^3$H]-$PGE_2$ was added as a tracer to evaluate procedural losses. PGs were extracted with a petroleum ether/diethyl ether mixture as described earlier (Karmali *et al.*, 1982). The organic extracts were then dried under nitrogen and taken up in buffer for subsequent measurement by RIA for $PGE_1$, $PGE_2$, $PGF_{2\alpha}$, 6-keto-$PGF_{1\alpha}$ and $TXB_2$.

*Statistical analysis* The $\log_{10}$ transformation was applied to tissue PG yields to make the statistical distributions more nearly Gaussian in order to apply classical statistical techniques (Student's paired or two-sample *t*-test, Pearson correlation and analysis of variance). Similarly, tumour size was transformed logarithmically, and the proportion of positive nodes was logistically transformed, log (No. of positive nodes + 1/6)/(No. of negative nodes + 1/6). Microsomal PG synthesis values were not transformed. The results of parametric tests were confirmed by their nonparametric counterparts (Wilcoxon signed rank test, Mann-Whitney U-test and Kendall rank correlation) used on the raw values. Student's *t*-test was used to compare two groups (e.g., distant metastasis present or absent) and analysis of variance was applied to test the relationship with Stage of disease (I, II vs III vs IV). Marginally significant results must be confirmed on a larger data set in view of the problem of multiplicity, i.e., simultaneous statistical testing of the 5 PGs with respect to each of the other variables. However, results of $P < 0.01$ are significant even by the conservative Bonferroni criterion.

### Results

Tissue yields and *in vitro* production of 5 prostanoids were measured in 24 neoplastic breast lesions and their associated noncancerous ductal tissues. These lesions were randomly obtained from breast masses removed surgically at Memorial Hospital.

*Tissue yields of prostaglandins and thromboxane B2 in noncancerous and neoplastic tissues*

The tissue yields (ng $g^{-1}$ wet wt) of all 5 immunoreactive compounds, $PGE_1$, $PGE_2$, $PGF_{2\alpha}$, 6-keto-$PGF_{1\alpha}$, and $TXB_2$, varied widely from lesion to lesion but the mean values were considerably higher in breast tumours than the associated noncancerous breast tissues. The following are ($\log_{10}$) values (ng $g^{-1}$ wet tissue): Mean ± s.e. with noncancerous values followed by tumour values: $PGE_1$: 0.557 ± 0.076 and 0.777 ± 0.097 ($P = 0.010$); $PGE_2$: 0.113 ± 0.157 and 0.470 ± 0.150 ($P = 0.032$); $PGF_{2\alpha}$: −0.330 ± 0.087 and 0.383 ± 0.120 ($P < 0.001$); 6-keto-$PGF_{1\alpha}$: −0.360 ± 0.141 and 0.398 ± 0.162 ($P < 0.001$); and $TXB_2$: −0.426 ± 0.103 and 0.186 ± 0.108 ($P < 0.001$).

Tumour and noncancerous tissue PG yields correlated significantly with each other ($P < 0.001$). Noncancerous tissue yields were thus used as controls to obtain "adjusted" values upon which the rest of the analysis was based.

The methodology has been tested carefully in our laboratory using mouse, rat and human mammary carcinomas to evaluate if PG production results during the processing of the material. Although this evaluation has involved processing of a large number of tumour specimens, data are presented for PG yield in 5 replicates of only one such tumour specimen processed at 2°C as described using MES buffer with or without ibuprofen (20 μg $ml^{-1}$). The following are mean values (ng $g^{-1}$ wet tissue) for control followed by samples processed in the presence of ibuprofen, both at 2°C: Mean ± s.e.: $PGE_1$: 150 ± 7.7. and 167 ± 11.9; $PGE_2$: 224 ± 30.6 and 192.1 ± 33.1; $PGF_{2\alpha}$: 8 ± 1.5 and 6 ± 0.4; 6-keto-$PGF_{1\alpha}$: 22 ± 7.1 and 28 ± 0; and $TXB_2$: 9 ± [illegible].1 and 10 ± 0.3. There was no significant difference in tumour yields between control samples and those processed in the presence of ibuprofen ($20\,\mu g\,ml^{-1}$).

The above test was repeated using 5 replicates of another tumour specimen and the extracted material was allowed to stand at room temperature for 60 min before centrifuging it. Tumour yields ($ng\,g^{-1}$ wet tissue) were lower in specimens that were processed in the presence of $20\,\mu g\,ml^{-1}$ ibuprofen in MES buffer: $PGE_1$: $49 \pm 8.1$ and $21 \pm 0$ ($P = 0.026$); $PGE_2$: $294 \pm 11.4$ and $166 \pm 91.2$; $PGF_{2\alpha}$: $43 \pm 6.3$ and $36 \pm 8.6$; 6-keto-$PGF_{1\alpha}$: $142 \pm 29.9$ and $51 \pm 15.8$ ($P = 0.054$); and $TXB_2$: $7 \pm 2.0$ and $5 \pm 2.0$. These results demonstrate the importance of maintaining the temperature at 2°C to prevent production of PGs during the processing of the material.

*Tissue prostaglandin and $TXB_2$ Yields: Tumour size* Adjusted $TXB_2$ yield (tumour-noncancerous levels) correlated with tumour size ($P = 0.04$) (Table II). There was no significant correlation between other prostanoids and tumour size.

**Table II** Association of adjusted tissue yields of prostaglandins and the tumour size or the proportion of positive axillary lymph nodes—Kendall Rank Correlation Coefficient

| | *$Log_{10}$(tumour—noncancerous)*[a] Tumour size ($n = 23$) | Positive nodes ($n = 24$) |
|---|---|---|
| $PGE_1$ | 0.073 | 0.175 |
| $PGE_2$ | 0.033 | 0.066 |
| $PGF_{2\alpha}$ | −0.024 | 0.109 |
| 6-keto-$PGF_{1\alpha}$ | 0.029 | 0.040 |
| $TXB_2$ | 0.195 ($P = 0.05$) | 0.327 ($P = 0.026$)[b] |

[a]Tumour-noncancerous represents the $\log_{10}$ PG levels (plus any metabolism during the tissue processing) in a breast tumour minus $\log_{10}$ PG amount measured in the non-cancerous breast tissue taken from the same breast mass. The units in this and subsequent tables are $\log_{10}$ $ng\,g^{-1}$ wet tissue. The non-cancerous specimen was collected by the nursing staff in the Pathology Laboratory under the supervision of the Clinical Pathology Fellow.

[b]A statistically significant correlation was noted between adjusted $TXB_2$ levels and the number of positive lymph nodes.

*Tissue prostaglandins and $TXB_2$ Yields: Proportion of positive axillary lymph nodes* The adjusted tumour $TXB_2$ levels correlated with the proportion of positive lymph nodes ($P = 0.026$). Adjusted tumour $TXB_2$ yields were calculated by comparing $TXB_2$ amounts from tumour tissue with those found in associated specimens of noncancerous breast tissue (Table II). Amounts of $PGE_1$, $PGE_2$, and $PGF_{2\alpha}$ and 6-keto-$PGF_{1\alpha}$ did not correlate statistically with the proportion of positive nodes.

*Tissue prostaglandin and $TXB_2$ yields: Metastases (bone, lung and liver)* There was no significant relationship between PG yields and presence of confirmed distant metastases (Table III).

**Table III** Adjusted tissue yields of prostaglandins and the spread of tumours to distant metastases ($n = 7$ +ve; $n = 17$ −ve)[a]

| | *$Log_{10}$ (tumour-noncancerous): Mean ±s.e.* + ve metastasis | − ve metastasis |
|---|---|---|
| $PGE_1$ | $-0.574 \pm 0.399$ | $-0.228 \pm 0.098$ |
| $PGE_2$ | $0.039 \pm 0.261$ | $-0.488 \pm 0.167$ |
| $PGF_{2\alpha}$ | $-0.581 \pm 0.187$ | $-0.706 \pm 0.125$ |
| 6-keto-$PGF_{1\alpha}$ | $-0.838 \pm 0.253$ | $-0.645 \pm 0.170$ |
| $TXB_2$ | $-0.509 \pm 0.163$ | $-0.565 \pm 0.147$ |

[a]There was no significant relationship between prostaglandin yields and presence of confirmed distant metastases.

*Tissue prostaglandin and $TXB_2$ yields: Oestrogen receptor positive or negative* When tumour PG yields were compared with respect to the oestrogen receptor content, the mean adjusted tumour $TXB_2$ yields (tumour-noncancerous) were significantly lower in receptor +ve tumours compared with receptor −ve ($P = 0.017$) (Table IV).

*Tissue prostaglandin and $TXB_2$ yields: Progesterone receptor content* Adjusted tumour 6-keto-$PGF_{1\alpha}$ yields were lower in progesterone +ve than in progesterone −ve receptor lesions ($P = 0.066$).

*Tissue prostaglandin and $TXB_2$ yields: Stage of malignancy* When tumour amounts of 5 compounds were analysed (without adjusting by subtracting the amounts from paired noncancerous tissue) in tumours with regard to Stage, $PGE_2$ tended to be higher ($P = 0.058$) and 6-keto-$PGF_{1\alpha}$ was lower ($P = 0.009$) with advancing Stage. These results are presented without subtracting the noncancerous values, for comparison with earlier studies by Rolland *et al.* (1980). Data of $PGE_2$ and 6-keto-$PGF_{1\alpha}$ have been presented in Table V; there was no significant difference in $PGE_1$, $PGF_{2\alpha}$ and $TXB_2$.

*Characterization of microsomal prostaglandin synthetase activity in noncancerous and neoplastic breast tissue*

The microsomal enzyme obtained from tumour and noncancerous homogenates generated ($ng\,mg^{-1}$ protein/10 min, Mean ±s.e.): $23.5 \pm 3.04$ and $25.5 \pm 3.48$ $PGE_1$; $16.5 \pm 2.92$ and $14.5 \pm 2.56$ $PGE_2$; $1.4 \pm 0.36$ and $3.3 \pm 1.51$ $PGF_{2\alpha}$; $8.0 \pm 1.78$ and 7.4

**Table IV** Adjusted tissue yields of prostaglandins and oestrogen or progesterone receptor content

| | *Oestrogen +ve* | | *Oestrogen −ve* | *Progesterone +ve* | | *Progesterone −ve* |
|---|---|---|---|---|---|---|
| | *$Log_{10}$ (tumour-noncancerous) PG content: Mean ±s.e.* | | | | | |
| *n* | 11 | | 13 | 6 | | 18 |
| $PGE_1$ | −0.504±0.260 | | −0.181±0.109 | −0.736±0.468 | | −1.932±0.081 |
| $PGE_2$ | −0.315±0.214 | | −0.351±0.208 | −0.573±0.350 | | −0.255±0.158 |
| $PGF_{2\alpha}$ | −0.573±0.127 | | −0.751±0.156 | −0.670±0.143 | | −0.669±0.130 |
| 6-keto-$PGF_{1\alpha}$ | −0.745±0.190 | | −0.664±0.207 | −1.064±0.177 | ($P$=0.066)[a] | −0.580±0.169 |
| $TXB_2$ | −0.850±0.187 | ($P$=0.017)[b] | −0.294±0.091 | −0.862±0.329 | | −0.445±0.098 |

[a]Tumour 6-keto-$PGF_{1\alpha}$ yields were lower than in non-cancerous tissue in both progesterone +ve and −ve receptor lesions; however, this difference was greater in the progesterone +ve receptor lesions.

[b]Tumour $TXB_2$ levels were lower relative to non-cancerous tissue in oestrogen +ve receptor breast lesions in comparison with receptor −ve lesions.

**Table V** Tissue yields of prostaglandins and stage of the breast malignancy

| | | *Stage I & II ($n$=8)* | | *Stage III ($n$=8)* | *Stage IV ($n$=7)* |
|---|---|---|---|---|---|
| | | *Log PG (mean ±s.e.)*[a] | | | |
| $PGE_2$ | Tumour: | 0.0364±0.2152 | ($P$=0.577)[b] | −0.2614±0.2288 | 0.6924±0.2390 |
| | Tumour-noncancerous: | −0.3638±0.2645 | | −0.6906±0.2199 | 0.0258±0.2629 |
| 6-keto-$PGF_{1\alpha}$ | Tumour: | 0.0907±0.2058 | ($P$=0.009)[c] | −0.1552±0.2321 | −0.7208±0.1031 |
| | Tumour-noncancerous: | −0.6335±0.2187 | | −0.5400±0.2438 | −1.0628±0.2700 |

[a]Data on $PGE_1$, $PGF_{2\alpha}$ and $TXB_2$ did not reach statistical significance; they will be provided on request.

[b]Tumour $PGE_2$ yields in Stage I and II breast lesions tended to be lower than the Stage IV lesions.

[c]Tumour 6-keto-$PGF_{1\alpha}$ yields decreased with advancing Stage of breast malignancy.

±1.49 6-keto-$PGF_{1\alpha}$ and 2.2±0.31 and 2.5±0.35 $TXA_2$, respectively. Enzyme activity was inhibited 58% when 20 $\mu g\,ml^{-1}$ ibuprofen was included in the incubation mixture. This suggests that much higher amounts of the inhibitor are required to bring about 100% inhibition. We have found this to be the case with mammary tumour cells *in vitro* where ibuprofen 100 $\mu g\,ml^{-1}$ of culture medium were required to prevent PG synthesis (Karmali and Cohen, unpublished observations). One feature of the PG synthetase is that while an agent causes a block in one organ, it will usually cause a block in another but there may be profound differences in the concentrations required (Flower, 1974; Flower & Vane, 1974).

Tumour and noncancerous tissue PG production *in vitro* correlated with each other: $PGE_1$ ($P$<0.001), $PGE_2$ ($P$<0.001), 6-keto-$PGF_{1\alpha}$ ($P$<0.001) and $TXB_2$ ($P$<0.001). Noncancerous tissue PG yields were thus used as controls to obtain "adjusted" values upon which the rest of the statistical analysis is based.

*PG synthetase activity: Tumour size* The only significant relationship between tumour size and adjusted PG production by microsomal PG synthetase was with $TXB_2$ ($P$=0.04) (Table VI).

**Table VI** Association of adjusted prostaglandin activity *in vitro* and tumour size or the proportion of positive axillary lymph nodes—Pearson Correlation Coefficient

| | *Tumour size ($n$=23)* | *Positive nodes ($n$=24)* |
|---|---|---|
| | *Tumour-noncancerous* | |
| $PGE_1$ | 0.025 | 0.118 |
| $PGE_2$ | −0.118 | −0.080 |
| $PGF_{2\alpha}$ | 0.094 | 0.149 |
| 6-keto-$PGF_{1\alpha}$ | 0.012 | −0.084 |
| $TXB_2$ | 0.308 ($P$=0.040)[a] | 0.356 ($P$=0.015)[b] |

[a,b]Adjusted tumour $TXB_2$ synthesis *in vitro* relative to non-cancerous tissue was significantly related to tumour size and number of positive nodes.

*PG synthetase activity: Proportion of positive axillary lymph nodes* The only significant correlation between the proportion of positive nodes and adjusted production by microsomal enzyme was with $TXB_2$ ($P$=0.015) (Table VI).

*PG synthetase activity: Distant metastases (to liver, bone and lung)* Seven lesions had detected positive distant metastases in the 24 patients studied. None of the adjusted tumour PG production yields were significantly different in patients with metastases compared with those not associated with detected metastases (Table VII).

**Table VII** Association of adjusted PG synthetase activity *in vitro* and metastasis of breast tumours to lung, bone and liver ($n=7$ +ve)[a]

| *Prostaglandin* | *Tumour noncancerous (mean ± s.e.)* + *ve metastasis* | − *ve metastasis* |
|---|---|---|
| $PGE_1$ | 23.51 ± 14.64 | 3.31 ± 15.72 |
| $PGE_2$ | −21.45 ± 44.66 | −19.59 ± 9.41 |
| $PGF_{2\alpha}$ | 2.75 ± 3.37 | 25.46 ± 20.61 |
| 6-keto-$PGF_{1\alpha}$ | −10.20 ± 18.96 | −5.22 ± 5.56 |
| $TXB_2$ | 5.19 ± 2.89 | 1.01 ± 1.30 |

[a]There was no significant relationship between PG synthetase activity *in vitro* and presence of confirmed distant metastases.

*PG synthetase activity: Oestrogen receptor content* Of the 24 breast masses, 11 were receptor +ve. Adjusted tumour $TXB_2$ production (tumour-noncancerous tissue) tended to be higher in receptor −ve breast lesions ($P=0.056$) (Table VIII).

*PG synthetase activity: Progesterone receptor content* Adjusted tumour $PGE_2$ production (tumour-noncancerous) in receptor +ve tumour was higher than in receptor −ve tumours ($P=0.046$) (Table VIII), whereas the reverse occurred with $TXB_2$ ($P=0.002$).

*PG synthetase activity: State of malignancy* There were no significant trends in adjusted microsomal PG synthesis *in vitro* with increasing Stage of breast malignancy.

### Discussion

While most of the previous studies in both human and experimental (Bennett *et al.*, 1975, 1977, 1979) mammary cancer have reported elevated $PGE_2$ tumour yields, this report demonstrates that other PG moieties such as $PGE_1$, $PGF_{2\alpha}$, $PGI_2$ and $TXA_2$ are formed by human mammary cancers. In addition, noncancerous tissue from the same resected specimen was studied in an attempt to compensate for individual variations and to standardise the methodology to evaluate the extent of the PG-related abnormality in the malignant breast specimen.

The mean amounts of PGs extracted from the breast tumours were considerably higher than those in paired noncancerous breast tissue controls. In studies of PGs in breast cancer reported by Kibbey *et al.* (1979), $PGE_2$ yields were higher than those measured in this study, possibly due to methodological differences.

The present results also show that microsomal enzyme fractions obtained from both noncancerous and neoplastic breast tissues can transform arachidonate (C20:4) to various prostanoids. With the exception of less $PGF_{2\alpha}$ synthesis by tumour microsomes, the mean production of the various PG moieties were comparable in both noncancerous and neoplastic breast tissues and were in the order $PGE_1 > PGE_2 >$ 6-keto-$PGF_{1\alpha} > TXB_2$ and $PGF_{2\alpha}$. The high production rates of $PGE_1$ by noncancerous and neoplastic microsomes was surprising because $PGE_1$ is synthesised from a

**Table VIII** Prostaglandin synthetase activity *in vitro* and oestrogen or progesterone receptor content

| | *Tumour-noncancerous (mean ± s.e.)* | | | | | |
|---|---|---|---|---|---|---|
| | *Oestrogen* + *ve* | | *Oestrogen* − *ve* | *Progesterone* + *ve* | | *Progesterone* − *ve* |
| $PGE_1$ | 2.29 ± 13.93 | | 14.96 ± 18.94 | −1.75 ± 24.63 | | 12.85 ± 13.90 |
| $PGE_2$ | −1.87 ± 15.86 | | −35.58 ± 21.70 | 22.10 ± 19.49 | ($P=0.046$)[b] | −34.20 ± 16.39 |
| $PGF_{2\alpha}$ | 2.64 ± 3.46 | | 32.54 ± 26.79 | 3.36 ± 5.80 | | 24.00 ± 19.44 |
| 6-keto-$PGF_{1\alpha}$ | −6.50 ± 6.69 | | −6.82 ± 10.95 | −4.92 ± 8.52 | | −7.26 ± 8.37 |
| $TXB_2$ | −0.39 ± 1.96 | ($P=0.056$)[a] | 4.45 ± 1.46 | −2.20 ± 0.71 | ($P=0.002$)[c] | 3.71 ± 1.54 |

[a]Tumour $TXB_2$ production (tumour-noncancerous tissue) tended to be higher in oestrogen −ve receptor breast lesions.

[b]Tumour $PGE_2$ production (tumour-noncancerous tissue) was higher in progesterone +ve receptor breast lesions.

[c]Tumour $TXB_2$ production (tumour-noncancerous tissue) was higher in progesterone −ve receptor breast lesions.

different precursor, dihomo-gamma-lino-lenate (C20:3), whereas the rest of the metabolites examined are from arachidonate, and this fatty acid was the only prostaglandin precursor added to the reaction mixture. Tests of cross-reactivity with $PGE_1$ antisera ruled out the possibility that $PGE_2$ was being measured instead of $PGE_1$.

The reason for the observed elevation in yields of immunoreactive PG-like material in breast cancer tissue is not clearly understood. Several proposed possibilities include: (1) increased enzyme synthetic activity; (2) decreased catabolic activity; (3) increased availability of precursor polyenoic acids; (4) a breakdown in the negative feedback controls which normally regulate PG formation (Horrobin, 1980).

In an attempt to evaluate the importance of each of the 5 PG moieties individually, we have analysed the relationships between tissue yields and PG production by microsomal PG synthetase with clinical variables such as tumour size, proportion of positive axillary lymph nodes, distant metastases (to liver, bone and lung), oestrogen receptor content, progesterone receptor content and Stage of breast malignancy. $TXB_2$ was the only arachidonate metabolite showing a significant relationship with tumour size and the number of positive nodes. However, tissue production of 6-keto-$PGF_{1\alpha}$ tended to be less in breast tumours associated with a higher number of positive nodes.

Rolland *et al.* (1980) concluded from a study of 91 breast leasions that $PGE_2$ production by microsomal PG synthetase may be used as a marker of high metastatic potential for neoplastic cells in breast cancer. Bennett *et al.* (1975, 1977) found that bone metastases were associated with tumours having high levels of PG-like material. Analysis without adjusting our $PGE_2$ results for the normal tissue values gives results consistent with these reports. In addition, we have adjusted our findings by subtracting the amount from the normal paired sample. Such an approach takes into consideration any variations due either to processing or to differences between individual specimens.

Several variables complicate the interpretation of PG studies in breast cancer masses. Mechanical disruption—a necessity in analysing PGs in solid tumours—can stimulate PG production but since ibuprofen did not alter the yield, it seems that disruption at 2°C does not stimulate PG synthesis by tumour tissue. Presumably normal tissues would not synthesise PGs with this method. Tissues are not homogenous and lymphocytes and monocytes are present to varying extents. They probably contribute to the PG yield but their numbers do not correlate to the amount of PG (Bennett *et al.*, 1977; Rolland *et al.*, 1980). PG production and metabolism may vary between individuals in a way unrelated to the neoplastic lesion. We have accounted for this in part by including a paired control of noncancerous breast tissue for each breast specimen. Further studies will be necessary to ascertain what enzymic defects or alterations may account for the excess PG tissue content characteristic of breast cancers.

Our preliminary results show that adjusted $TXA_2$ production correlated with tumour size, axillary lymph node metastases and distant metastasis, sometimes with a concomitant decrease in $PGI_2$. Honn *et al.* (1980, 1981, 1983) have proposed that the intravascular balance between $PGI_2$ and $TXA_2$ is disrupted in favor of platelet aggregation during development of tumour cell metastasis from Lewis lung carcinoma and B16 melanoma in mice. Such a shift in balance of $TXA_2/PGI_2$ in favor of $TXA_2$ was also found to favour metastasis in two metastatic variants of a murine fibrosarcoma (Donati *et al.*, 1982). These findings in experimental studies support our preliminary results and suggest that tumour $TXB_2$, $PGI_2$ and $PGE_2$ may be of value as tests for prognostic factors in breast cancer.

The help and cooperation from the staff of the Tumour Procurement Center, Sloan-Kettering Institute, is very much appreciated, with our special thanks to Lois Almadronos, Registered Nurse Coordinator.

This project was supported by the Special Projects Grant from Sloan-Kettering Institute and Grant PDT-208 from the American Cancer Society.

**References**

AMERICAN JOINT COMMITTEE. (1979). *Cancer Staging and End Results Reporting*. Chicago: Whiting Press, p. 108.

BENNETT, A. (1979). Prostaglandins and Cancer. In: *Practical Application of Prostaglandins and their Synthesis*, (Ed. Karim). p. 149.

BENNETT, A., McDONALD, A.M., SIMPSON, J.S. & STAMFORD, I.F. (1975). Breast cancer, prostaglandins and bone metastases. *Lancet*, **i**, 1218.

BENNETT, A., CHARLIER, E.M., McDONALD, A.M., SIMPSON, J.S., STAMFORD, I.F. & ZEBRO, T. (1977). Prostaglandin and breast cancer. *Lancet*, **ii**, 624.

BENNETT, A., BERSTOCK, D.A., RAJA, B. & STAMFORD, I.F. (1979). Survival time after surgery is inversely related to the amounts of prostaglandins extracted from human breast cancers. *Br. J. Pharmac.*, **66**, 451P.

DONATI, M.B., BOROWSKA, A., BOTTAZZI, B. & 4 others. (1982). *Metastatic Potential Correlates with Changes in the Thromboxane-prostacyclin Balance*. Presented at V International Conference on Prostaglandin May 1982, Florence, Abstr. 136.

FLOWER, R.J. (1974). Drugs which inhibit prostaglandin synthesis. *Pharmacol. Rev.*, **26**, 33.

FLOWER, R.J. & VANE, J.R. (1974). Some pharmacologic and biochemical aspects of prostaglandin biosynthesis and its inhibition. In: *Prostaglandin Synthetase Inhibitors*, p. 9. (Eds. Robinson & Vane) Raven Press, New York.

HONN, K.V. (1980). Prostacyclin/thromboxane ratios in tumor growth and metastasis. *Cancer Res.*, **40**, 733.

HONN, K.V., CICONE, B. & SKOFF, A. (1981). Prostacyclin: A potent antimetastatic agent. *Science*, **212**, 1270.

HONN, K.V., BUESE, W.D. & SLOANE, B.F. (1983). Prostacyclin and thromboxanes. Implications for their role in tumor cell metastasis. *Biochem. Pharmacol.*, **32**, 1.

HORROBIN, D.F. (1980). The reversibility of cancer: The relevance of cyclic AMP, calcium, essential fatty acids and prostaglandin $E_1$. *Med. Hypothesis*, **6**, 469.

KARMALI, R.A. (1980). Review: Prostaglandins and Cancer. *Prostaglandins Med.*, **5**, 11.

KARMALI, R.A., MUSE, P., ALLEN, G. & LOUIS, T. (1982). Macrophage production of prostaglandins: Effects of foetal calf serum and diazepam: Use of an improved method for extracting 6-keto-$PGF_{1\alpha}$. *Prostaglandins Leukotrienes Med.*, **8**, 565.

KIBBEY, W.F., BRONN, D.G. & MINTON, J.P. (1979). Prostaglandin synthetase and prostaglandin $E_2$ levels in human breast carcinoma. *Prostaglandins Med.*, **2**, 133.

ROLLAND, P.H., MARTIN, P.M., JACQUEMLER, J., ROLLAND, A.M. & TOGA, M. (1980). Prostaglandin in human breast cancer: Evidence suggesting that an elevated prostaglandin production is a marker of metastatic potential for neoplastic cells. *J. Natl Cancer Inst.*, **61**, 1061.

SEYBERTH, H.W., SEGRE, G.V., MORGAN, J.L., SWEETMAN, B.J., POTTS, J.T. & OATES, J.A. (1975). Prostaglandins as mediators of hypercalcemia associated with certain types of cancer. *N. Engl. J. Med.*, **293**, 1278.


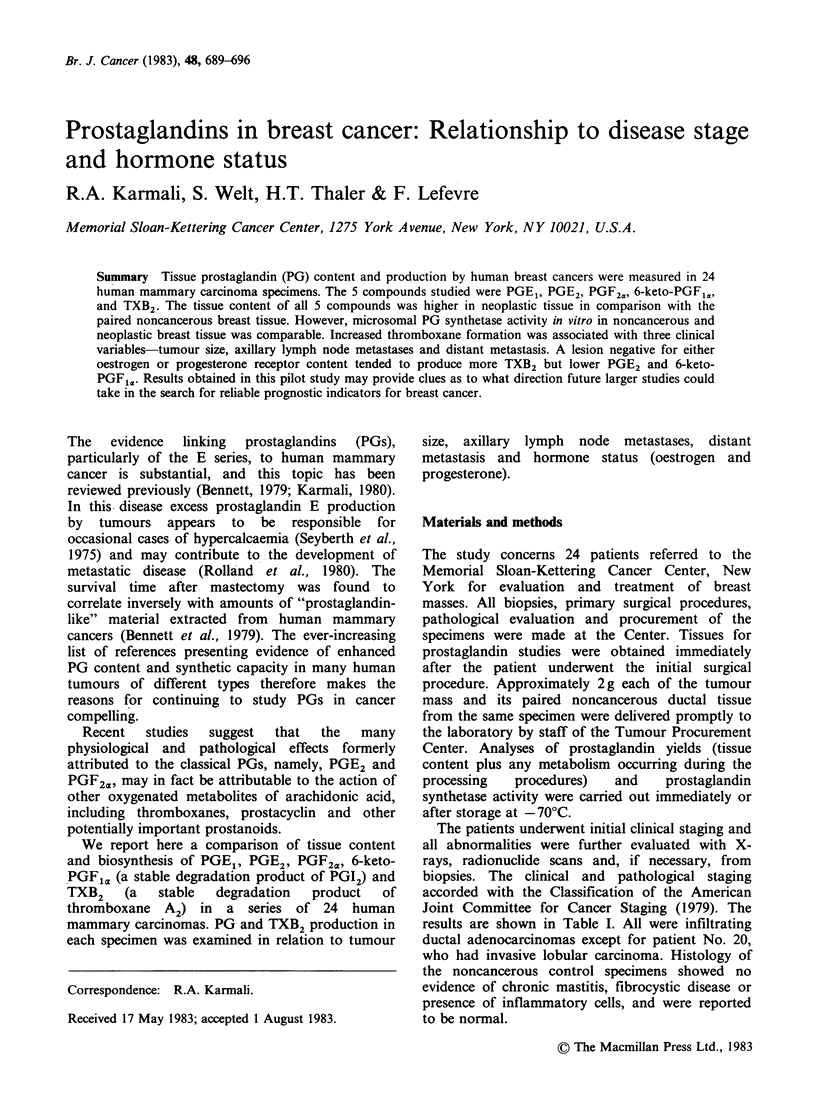

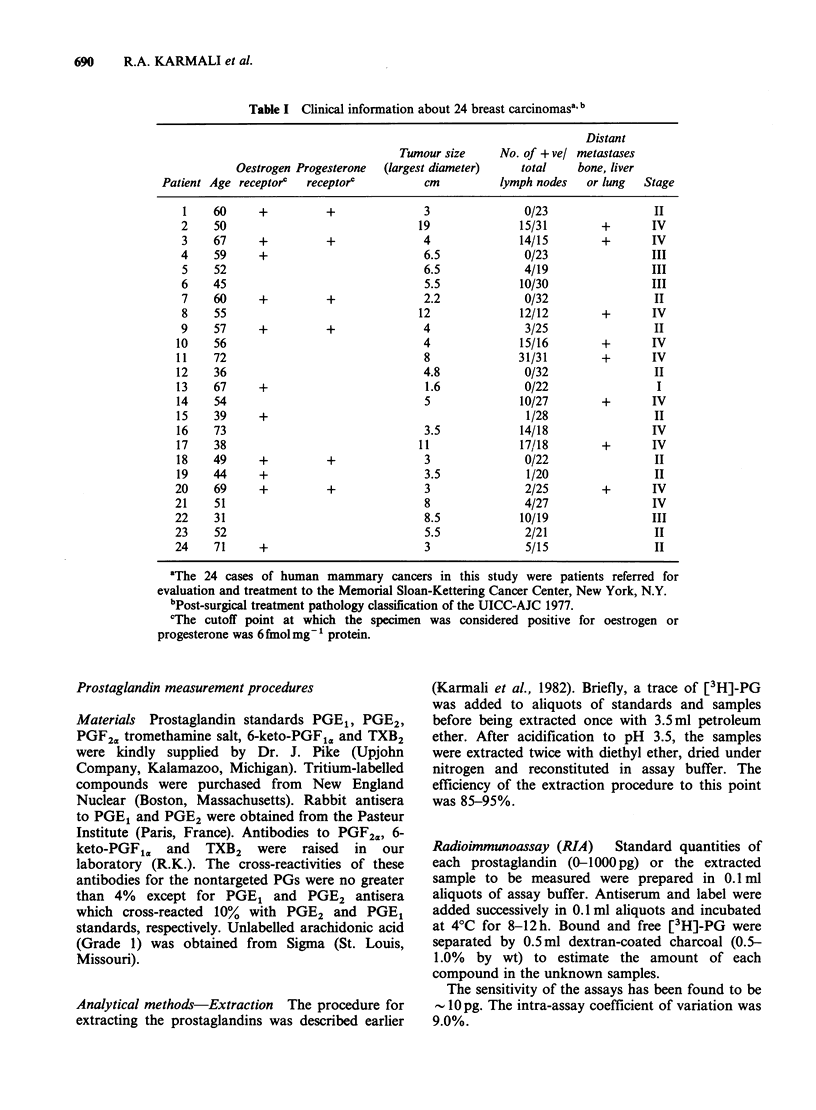

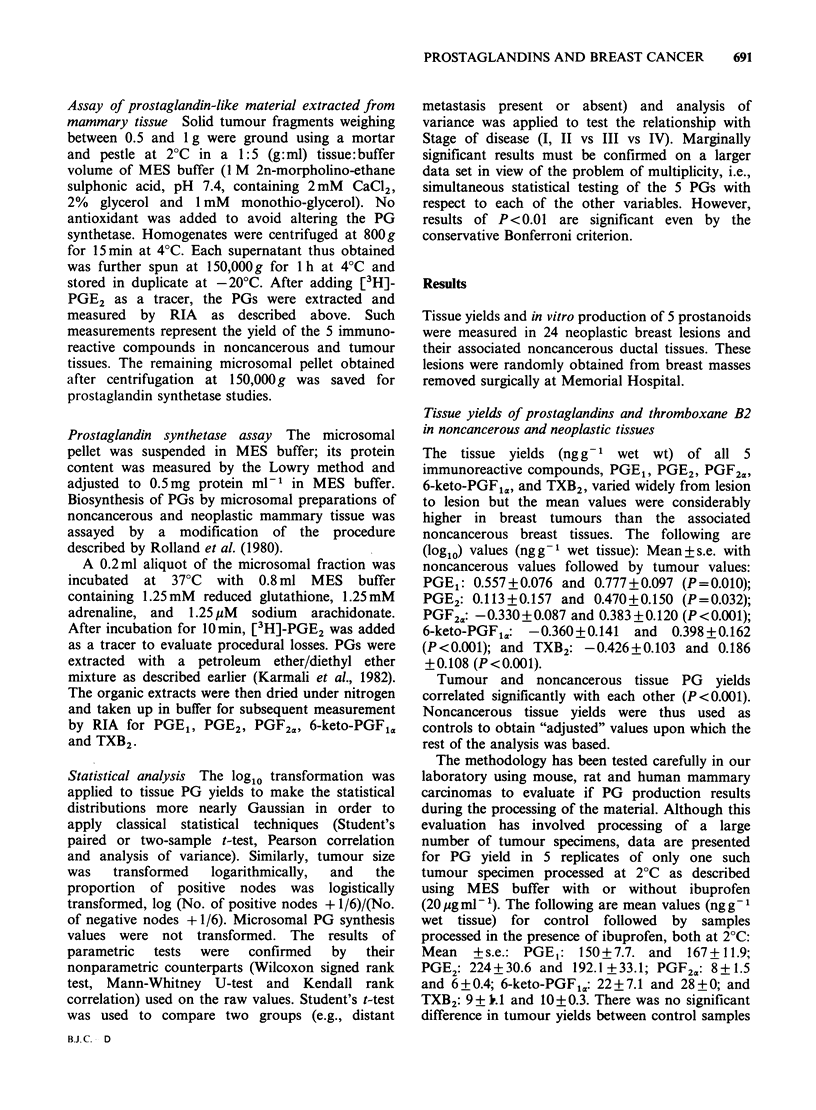

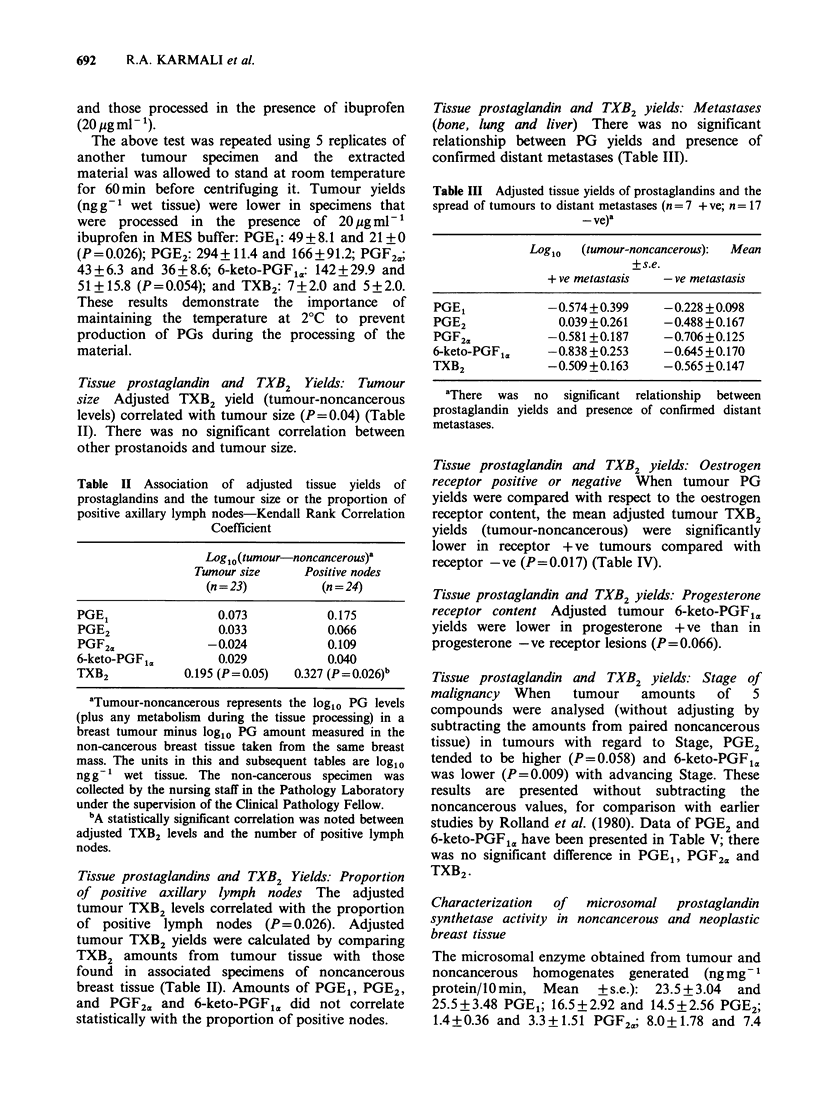

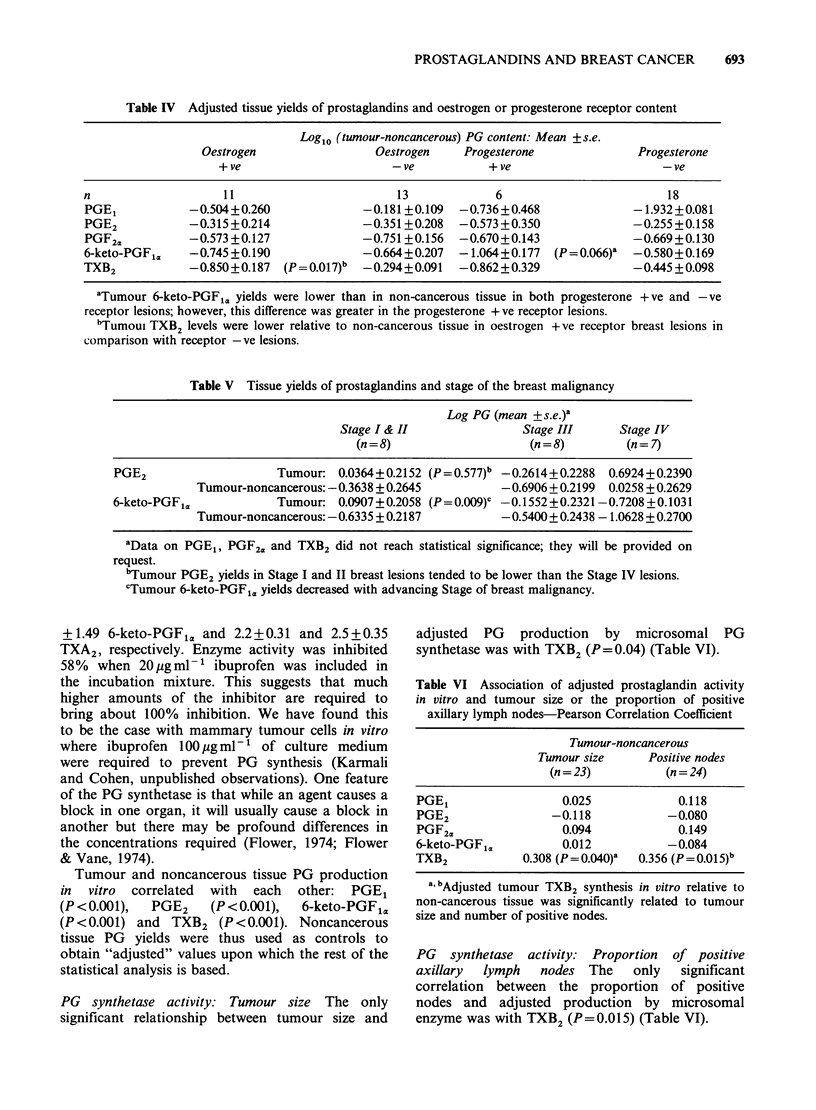

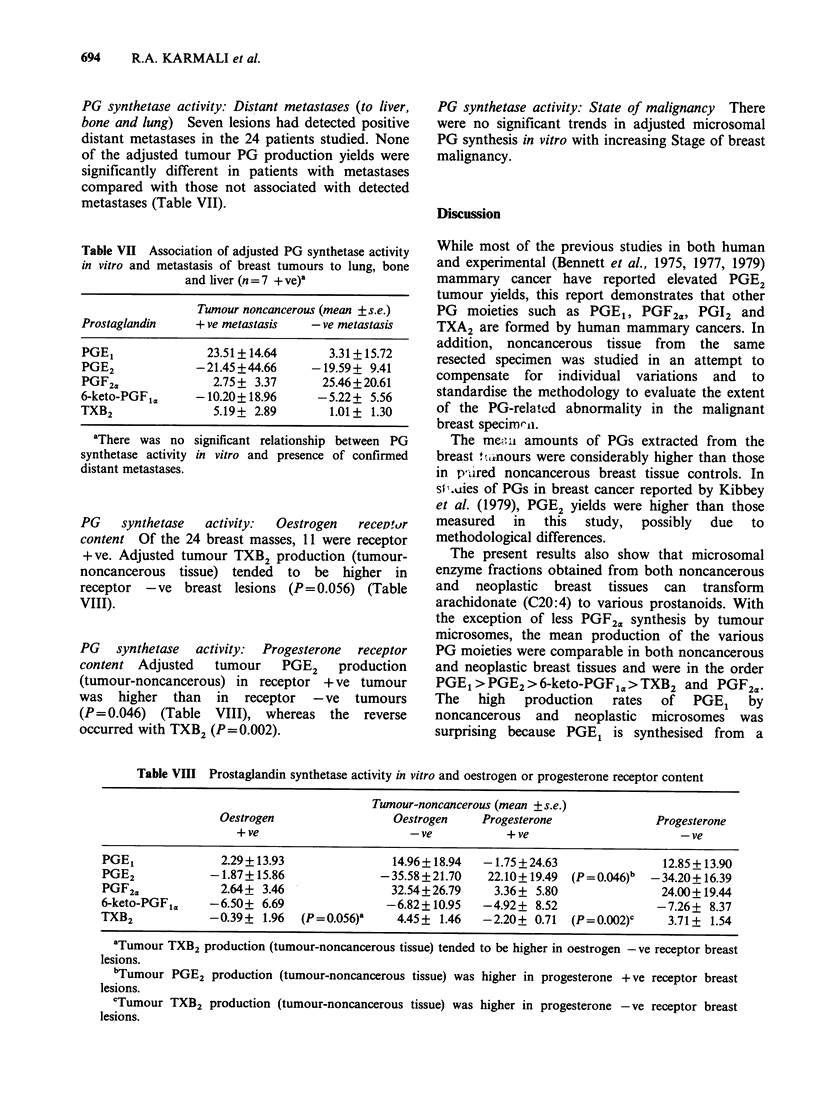

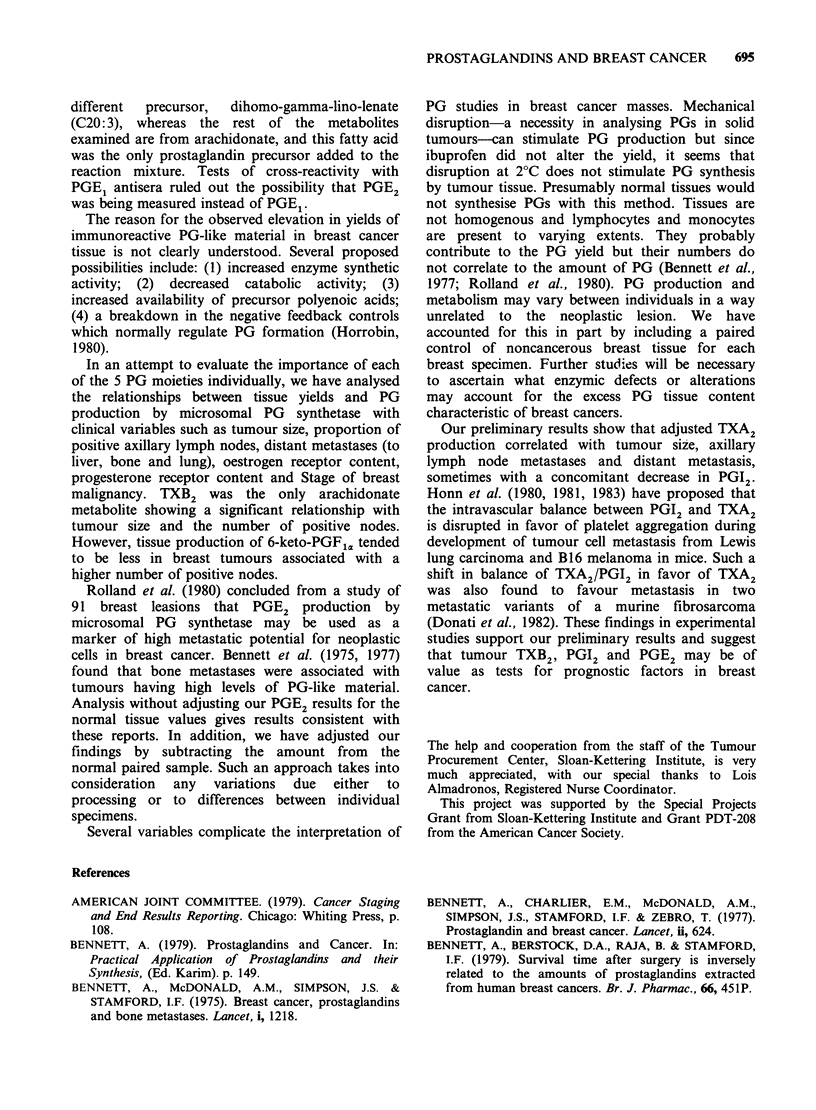

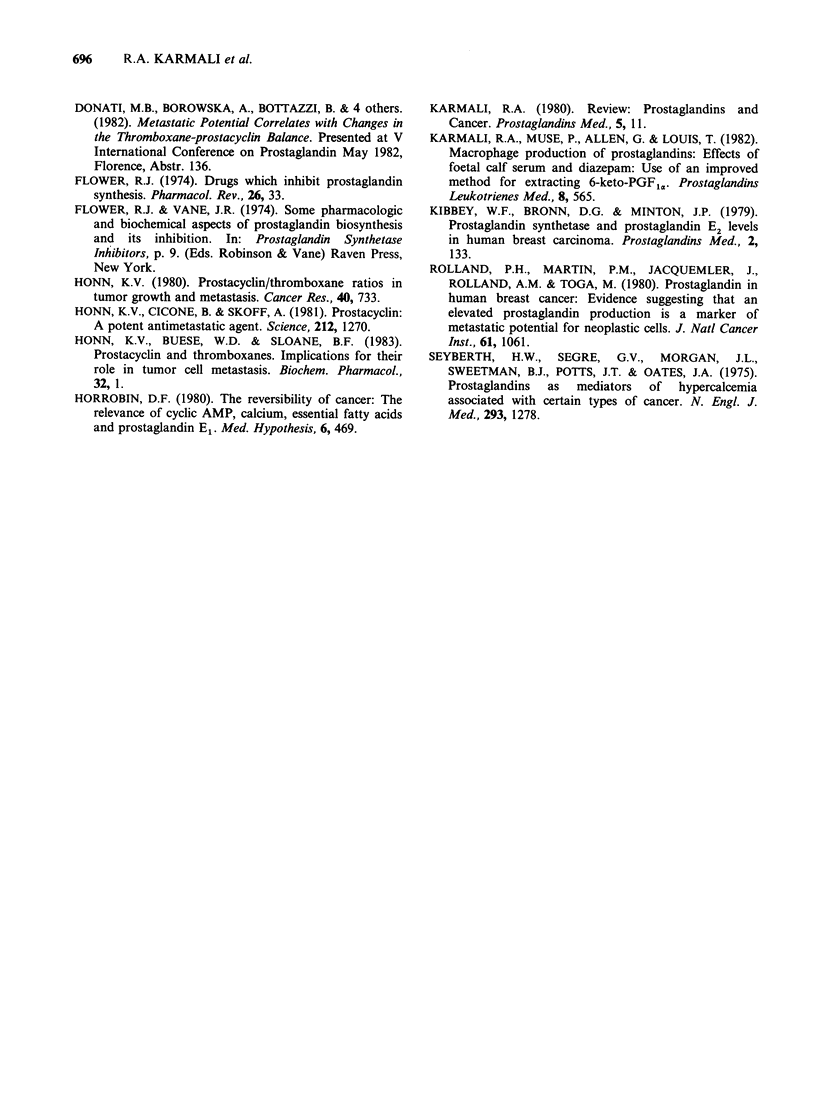


## References

[OCR_00796] Bennett A., Berstock D. A., Raja B., Stamford I. F. (1979). Survival time after surgery is inversely related to the amounts of prostaglandins extracted from human breast cancers [proceedings].. Br J Pharmacol.

[OCR_00791] Bennett A., Charlier E. M., McDonald A. M., Simpson J. S., Stamford I. F., Zebro T. (1977). Prostaglandins and breast cancer.. Lancet.

[OCR_00786] Bennett A., McDonald A. M., Simpson J. S., Stamford I. F. (1975). Breast cancer, prostaglandins, and bone metastases.. Lancet.

[OCR_00811] Flower R. J. (1974). Drugs which inhibit prostaglandin biosynthesis.. Pharmacol Rev.

[OCR_00830] Honn K. V., Busse W. D., Sloane B. F. (1983). Prostacyclin and thromboxanes. Implications for their role in tumor cell metastasis.. Biochem Pharmacol.

[OCR_00826] Honn K. V., Cicone B., Skoff A. (1981). Prostacyclin: a potent antimetastatic agent.. Science.

[OCR_00836] Horrobin D. F. (1980). The reversibility of cancer: the relevance of cyclic AMP, calcium, essential fatty acids and prostaglandin E1.. Med Hypotheses.

[OCR_00845] Karmali R. A., Muse P., Allen G., Louis T. (1982). Macrophage production of prostaglandins: effects of fetal calf serum and diazepam. Use of an improved method for extracting 6-keto-PGF1 alpha.. Prostaglandins Leukot Med.

[OCR_00841] Karmali R. A. (1980). Review: prostaglandins and cancer.. Prostaglandins Med.

[OCR_00852] Kibbey W. E., Bronn D. G., Minton J. P. (1979). Prostaglandin synthetase and prostaglandin E2 levels in human breast carcinoma.. Prostaglandins Med.

[OCR_00858] Rolland P. H., Martin P. M., Jacquemier J., Rolland A. M., Toga M. (1980). Prostaglandin in human breast cancer: Evidence suggesting that an elevated prostaglandin production is a marker of high metastatic potential for neoplastic cells.. J Natl Cancer Inst.

[OCR_00866] Seyberth H. W., Segre G. V., Morgan J. L., Sweetman B. J., Potts J. T., Oates J. A. (1975). Prostaglandins as mediators of hypercalcemia associated with certain types of cancer.. N Engl J Med.

